# Allergen-specific IgE: comparison between skin prick test and serum assay in real life 

**DOI:** 10.5414/ALX01891E

**Published:** 2019-12-30

**Authors:** D. Bignardi, P. Comite, I. Mori, F. Ferrero, V. Fontana, M. Bruzzone, M. Mussap, G. Ciprandi

**Affiliations:** 1 Allergy Department,; 2 Laboratory Medicine, and; 3 Clinical Epidemiology Unit, IRCCS-AOU San Martino, Genoa, Italy

**Keywords:** allergic rhinitis, asthma, conjunctivitis, serum IgE, skin prick test, real life

## Abstract

Background: The most common sensitizing allergens in in the area of Liguria region (Northwestern Italy) are pollens, mainly *Parietaria* and cypress, house dust mites, i.e. *Dermatophagoides*, and pets. IgE assessment is a crucial step in allergy diagnosis. It may be performed by skin prick test (SPT) or serum IgE (sIgE) assay. Therefore, this study compared these two methods in a real-life setting. Methods: This retrospective study included 793 subjects, who were referred to the Allergy Department for respiratory allergy during 2014. Inclusion criteria were i) documented diagnosis of allergic rhinitis (AR), and/or allergic asthma, and/or allergic conjunctivitis. SPT and sIgE assay were performed for 5 allergens, such as *Dermatophagoides pteronyssinus* (D1), cat (E1), *Parietaria officinalis* (W19), cypress (T23), and dog (E5), as they are the most common in our geographic area. Results: Using a positive SPT result as the target condition, remarkably high and statistically significant values of AUC, ranging from 0.84 to 0.94, were found. On the basis of the Youden index the following optimal classification threshold values were also computed: D1 = 0.22, E1 = 0.26, W19 = 0.61, T23 = 0.25, E5 = 0.34. These values allowed to define a set of sensitivity/specifity estimates ranging from 0.75 to 0.93 and from 0.83 to 0.93, respectively. Conclusions: The present study shows that SPT and sIgE are two tests that are rather concordant, but with different sensitivity and specificity distinct for each allergen. In clinical practice, both tests should be used depending on clinical history features and obtained findings.

**German version published in Allergologie, Vol. 40, No. 1/2017, pp. 16-22**

## Introduction 

Allergic disorders, mainly respiratory allergy, such as allergic rhinitis (AR), asthma (AA), and conjunctivitis (AC), are very common, as their prevalence may be up to 40% of the general population [1, 2]. The hallmark of the immune response in allergic patients is the ongoing production of allergen-specific IgE. This phenomenon is defined as sensitization. Sensitization can be considered the conditio sine qua non for diagnosing allergic disorders. On the other hand, the natural history of allergy is frequently characterized by an increasing number of sensitizations (such as the polysensitization phenomenon). In fact, atopic infants often start with mono-sensitization (sensitization to one single allergen), but quite soon tend to become sensitized to other allergens over time [3, 4]. Polysensitization is an immunological event that is relevant from an epidemiological and clinical point of view, as the prevalence ranges from 20% – 90%, with a great variability depending on the investigated population [5, 6, 7]. 

Sensitization can be demonstrated in vivo, by skin prick test (SPT) or in vitro, by serum IgE (sIgE) assay. In many countries, SPT is considered the first line diagnostic method, and sIgE assay as second line. SPT is usually performed using commercially available allergen extracts [8]. Sometimes prick-to-prick testing may be performed stinging the native substance and subsequently the skin. sIgE assay is usually performed using immune-enzymatic methods [9, 10]. However, both methods may have pros and cons. SPT is cheap, quick, and sensitive, sIgE assay is considered to be more specific, but more expensive and results are not immediate. The comparison between the two methods has been evaluated in some studies, but the findings may not always be reliable in all settings because of relevant environmental differences that affect the sensitization patterns [11, 12, 13, 14, 15, 16, 17, 18, 19]. 

The most common sensitizing allergens in the area of Liguria (Northwestern Italy) are: pollens, mainly *Parietaria officinalis* and cypress, house dust mites, especially *Dermatophagoides*, and pets [20]. Therefore, this study compared the SPT with the sIgE assay in a real-life study including patients with AR and/or AA and/or AC. 

## Material and methods 

Overall, 793 patients who visited a third level Allergy Department for espiratory allergy (mean age/range: 40.4, 6.0 – 85.0 years; males/%: 280/35) were involved in this study and 5 allergens were considered for analysis, specifically: *Dermatophagoides pteronyssinus* (D1), cat (E1), *Parietaria officinalis* (W19), cypress (T23), and dog (E5), as they are the most common in our geographic area. 

Diagnosis of AR, AA, and AC was documented by the doctor who initiated allergy testing and performed by validated criteria [1, 2, 3, 4, 5, 6, 7, 8, 9, 10, 11, 12, 13, 14, 15, 16, 17, 18, 19, 20, 21]. 

SPT was performed as stated by the European Academy of Allergy and Clinical Immunology [22]. The panel consisted of: house dust mites (*Dermatophagoides farinae* and *pteronyssinus*), cat, dog, grasses mix, Compositae mix, *Parietaria officinalis*, cypress, birch, hazel, olive tree, *Alternaria tenuis*, *Cladosporium*, Aspergilli mix (Stallergenes, Milan, Italy). 

Serum levels of specific IgE were detected by the IFMA procedure (ImmunoCAP Thermo Fisher Scientific, Uppsala, Sweden) in peripheral blood samples from patients. Serum was collected into gel-separator tubes, centrifuged, and stored at –20 °C until analysis. Measurement of circulating specific IgE antibodies was performed according to manufacturer’s instructions [23]. Specific IgE levels were expressed in kUA/L according to the traceable calibration to the 2^nd^ IRP WHO for Human IgE, and 0.35 kUA/L was the cut-off-value [24]. 

Statistical analysis was performed following these criteria: distributions of sIgE levels by sex, age, and time at blood sample were graphically explored using histograms, box plots, and QQ plots. Given the positive skewness of sIgE levels, data were log-transformed and described using geometric mean (GM), median (P50) and inter-quartile range (IQR). 

The classification performance of each allergen-specific sIgE was evaluated through receiver operating characteristic (ROC) analysis, assuming, a positive SPT result as the indicator of a true target condition. In particular, area under the ROC curve (AUC) was used as an estimate of the overall correct classification (accuracy) of enrolled patients, and the Youden index (YI) as a statistical criterion to define optimal classification threshold (OCT), namely allergen-specific sIgE values capable of minimizing false classification probabilities. According to the OCT value of each allergen-specific sIgE and SPT results, sensitivity (Se), i.e, the proportion of patients above the OCT with the true target condition (SPT positives), and specificity (Sp), i.e, the proportion of patients under the OCT without the true target condition (SPT negatives), were computed [25]. In addition, the joint effect of all health conditions considered in this analysis (i.e., rhinitis, asthma, and conjunctivitis) on SPT result and sIgE levels was estimated through regression modeling. Specifically, logistic regression was applied to SPT dichotomous outcome, while lognormal regression was used to model sIgE levels. In both cases, relative indexes of effect were computed: odds ratio (OR) in logistic modeling and geometric mean ratio (GMR) in log-normal modeling [26]. In other words, all variables were considered to define the probability of the expected outcomes. These indexes can be interpreted as ratios between the risk of being allergic among patients with airway and/or eye symptoms and the analogous risk among patients without symptoms. In all modeling, gender, age, month (season) and year of examination were taken into consideration as confounding variables. 

All statistical indexes calculated in this investigation were provided with 95% confidence limits (95% CL), and a p-value < 0.05 was considered as statistically significant. 

All data were analyzed using Stata statistical package version 13.1 (StataCorp, College Station, TX. USA). 

## Results 

Overall, 794 patients (male/female: 280/513; mean age/range: 40.4/6 – 85 years) were considered for analysis. Table 1 reports some results of exploratory analyses: D1 is the most relevant sensitizing allergen at SPT (54.4% of tested patients were sensitized), followed by E1 (33.2%), W19 (32.4%), T23 (26.8%), and E5 (22.8%). Superimposable percentages of sensitization were detected at sIgE assay: D1 = 55.1%; E1 = 35.3%; W19 = 35.7%; T23 = 29.4%; and E5 = 30.8%. The sIgE levels are reported in Table 1. 

In addition, Table 2 shows the distribution of patients affected by rhinitis and/or asthma and/or conjunctivitis according to the 5 tested allergens. The highest prevalence rates were observed in AC patients followed by AA patients and AR patients. 

**Figure 1.**
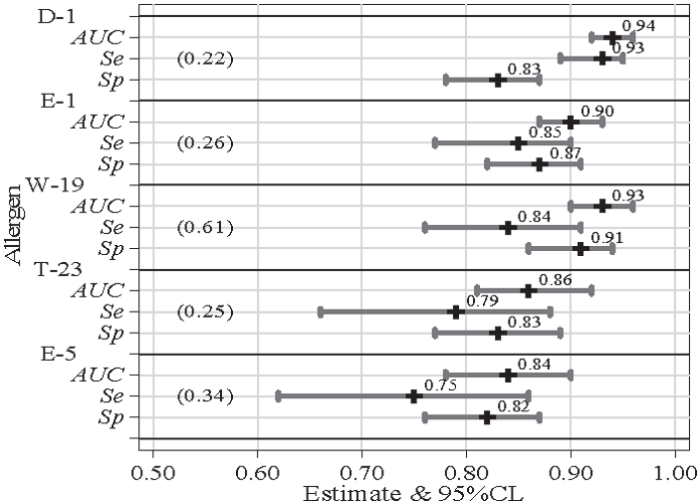
Point and interval estimates of serum IgE classification performance parameters according to skin prick test result (positive vs. negative). In parenthesis the optimal classification threshold, derived from the Youden index. AUC = area under ROC curves; Se = sensitivity; Sp = specificity.

Classification accuracy of all allergen-specific sIgE assays assessed through ROC analysis is summarized in Figure 1. Using SPT result as the target condition, remarkably high and statistically significant values of AUC, ranging from 0.84 to 0.94, were found. On the basis of the YI, the following OCT values were also computed: D1 = 0.22, E1 = 0.26, W19 = 0.61, T23 = 0.25, E5 = 0.34. These values allowed to define a set of Se/Sp estimates ranging from 0.75 – 0.93 and from 0.83 – 0.93, respectively. 

**Figure 2.**
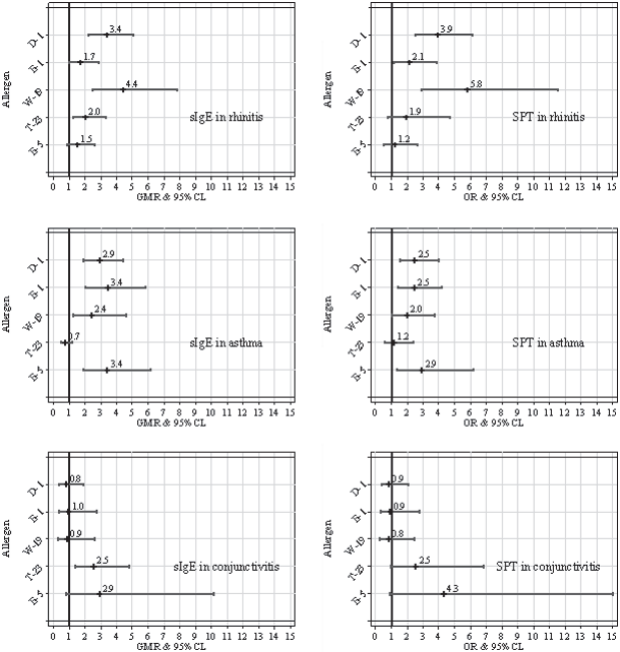
Joint effect of rhinitis, asthma, and conjunctivitis on allergen-specific serum IgE (sIgE) levels and skin prick test (SPT) result (positive vs. negative) estimated through log-normal (left column) and logistic (right column) regression modeling. All regression results are adjusted for gender, age, month, and year of examination. GMR = geometric mean ratio; OR = odds ratio; 95% CL = 95% confidence limits for GMR/OR.

Figure 2 depicts the joint effect of rhinitis, asthma and conjunctivitis on sIgE levels (left column) and STP outcome (right column). After adjusting for gender, age, month, and year of examination, similar allergenic risk patterns were pointed out within each health condition, although some discrepancies in allergic risk ratio (GMR and OR) can be found as a consequence of the different regression modeling adopted owing to the different outcome available for analysis (continuous for sIgE assay, dichotomous for SPT). 

## Discussion 

Allergic disorders are very common and their management represents an important burden for Health Service worldwide, both concerning the diagnostic approach and the treatment. Moreover, adequate treatment of allergic rhinitis, allergic conjunctivitis or mild allergic bronchial asthma is based on allergen immunotherapy with the administration of the causal allergen. Thus, appropriate allergy diagnosis is mandatory. In this regard, the documentation of IgE production, i.e. sensitization, is the main step. Sensitization may be evaluated by in-vivo testing, such as SPT, and/or in vitro testing, such as sIgE assay. 

The present study was aimed to compare SPT with sIgE assay in a real-life setting, enrolling consecutive patients who visited a third level Allergy Department for respiratory allergy. 

The findings show that both methods are reliable and substantially are superimposable, even though sensitivity and specificity are different considering the single test and the single allergen. 

This outcome underlines two main concepts:

- SPT may be considered a first-level approach.
- sIgE assay should be performed when SPT is not sufficient for allergy diagnosis.

On the other hand, the present study has some limitations: the presence of allergy was not confirmed by challenge tests, SPT was considered as real marker of allergy, which is not always the case. Moreover, subgroups were not homogeneous, and polysensitization as well as polyallergy were not evaluated. Therefore, further studies should be conducted to evaluate both tests in patients with confirmed allergy. 

In conclusion, the present study shows that SPT and sIgE are two tests that are rather concordant, but with different sensitivity and specificity distinct for each allergen. In clinical practice, both tests could be used depending on clinical history and obtained findings. 

## Conflict of interests 

The study had no funding source. The co-authors have no financial disclosure and conflict of interest. 

**Table 1.** Descriptive analysis of serum IgE (sIgE) levels and skin prick test (SPT) result according to allergens considered for analysis.

**Allergy test**	**Index**	**Allergen**				
		**D1**	**E1**	**W19**	**T23**	**E5**
	N	594	371	333	228	250
	GM	1.01	0.31	0.46	0.22	0.26

**sIgE assay**	95%CL	0.83-1.23	0.25-0.39	0.36-0.59	0.19-0.27	0.21-0.33
	P50	0.565	0.04	0.06	0.08	0.06
	IQR	0.04-7.51	0.00-1.37	0.02-2.05	0.02-0.59	0.01-0.62
	N (%) > 0.35	327 (55.1)	131 (35.3)	119 (35.7)	67 (29.4)	77 (30.8)

**SPT positivity**	N (%)	323 (54.4)	123 (33.2)	108 (32.4)	61 (26.8)	57 (22.8)

N = number of patients tested; GM = geometric mean; 95%CL = 95% confidence limits of GM;

**Table 2.** Number (N) and percentage (%) of patients affected by rhinitis and/or asthma and/or conjunctivitis according to allergens considered for analysis.

**Health condition**	**Allergen**									
	**D1**		**E1**		**W19**		**T23**		**E5**	
	N	(%)	N	(%)	N	(%)	N	(%)	N	(%)
Rhinitis	193	(36.2)	123	(37.9)	114	(39.9)	61	(30.1)	84	(39.6)
Asthma	389	(73.0)	230	(70.8)	223	(78.0)	145	(71.4)	150	(70.8)
Conjunctivitis	506	(94.9)	308	(94.8)	269	(94.1)	182	(89.7)	208	(96.2)
